# IPD 2.0: To derive insights from an evolving *SARS-CoV-2* genome

**DOI:** 10.1186/s12859-021-04172-x

**Published:** 2021-05-13

**Authors:** Sanket Desai, Aishwarya Rane, Asim Joshi, Amit Dutt

**Affiliations:** 1 Integrated Cancer Genomics Laboratory, Advanced Centre for Treatment, Research, and Education in Cancer, Tata Memorial Centre, Kharghar, Navi Mumbai, Maharashtra 410210 India; 2 Homi Bhabha National Institute, Training School Complex, Anushakti Nagar, Mumbai, Maharashtra 400094 India; 3 Adjunct Faculty, Institute of Advanced Virology, Kerala State Council for Science, Technology and Environment, Govt. of Kerala, Thonnakkal, Kerala 695317 India

**Keywords:** *SARS-CoV-2*, Pathogen analysis pipeline, Phylogenetic clade analysis, Next-generation sequencing

## Abstract

**Background:**

Rapid analysis of *SARS-CoV-2* genomic data plays a crucial role in surveillance and adoption of measures in controlling spread of Covid-19. Fast, inclusive and adaptive methods are required for the heterogenous *SARS-CoV-2* sequence data generated at an unprecedented rate.

**Results:**

We present an updated version of the *SARS-CoV-2* analysis module of our automated computational pipeline, Infectious Pathogen Detector (IPD) 2.0, to perform genomic analysis to understand the variability and dynamics of the virus. It adopts the recent clade nomenclature and demonstrates the clade prediction accuracy of 92.8%. IPD 2.0 also contains a *SARS-CoV-2* updater module, allowing automatic upgrading of the variant database using genome sequences from GISAID. As a proof of principle, analyzing 208,911 *SARS-CoV-2* genome sequences, we generate an extensive database of 2.58 million sample-wise variants. A comparative account of lineage-specific mutations in the newer *SARS-CoV-2* strains emerging in the UK, South Africa and Brazil and data reported from India identify overlapping and lineages specific acquired mutations suggesting a repetitive convergent and adaptive evolution.

**Conclusions:**

A novel and dynamic feature of the *SARS-CoV-2* module of IPD 2.0 makes it a contemporary tool to analyze the diverse and growing genomic strains of the virus and serve as a vital tool to help facilitate rapid genomic surveillance in a population to identify variants involved in breakthrough infections. IPD 2.0 is freely available from http://www.actrec.gov.in/pi-webpages/AmitDutt/IPD/IPD.html and the web-application is available at http://ipd.actrec.gov.in/ipdweb/.

**Supplementary Information:**

The online version contains supplementary material available at 10.1186/s12859-021-04172-x.

## Background

The *SARS-CoV-2* is mutating and evolving with time and geographical distribution, as typical of any RNA virus, indicating the generation of an increasing pool of emerging diversity in the viral strains [1]. The emergence of newer variants with higher infectivity or potential to impact vaccine efficacy underlines the significance of enhancing efforts to sequence the genome of the virus from across the globe. Genome sequencing of *SARS-CoV-2* is the most widely used method for tracking strains and identifying novel emerging variants in the population. Several national initiatives have enacted active genomic surveillance to identify novel region-specific variants involved in breakthrough infections [2]. Even a modest increase in infectivity rate of a regional variant or a reduction in vaccine efficacy or increased transmission would require immediate stringent measures to be put in place to contain the spread of the strain. Thus, automated measures are needed to perform integrated analysis to identify the newer variants.

We recently developed a computational tool, Infectious Pathogen Detector (IPD), with a *SARS-CoV-2* module to determine the abundance, mutation rate and phylogeny of the *SARS-CoV-2* genome from the heterogeneous advanced sequencing data [3]. In the current manuscript, we present its updated version (IPD 2.0), which adopts the evolving nomenclature of the *SARS-CoV-2* clades [4] and a *SARS-CoV-2* variant database updater module, allowing users to update the variants from viral strains with the inclusion of recently deposited strains in the GISAID database. This unique feature makes IPD 2.0 an adaptable tool for variant and clade analysis of the sequencing data from the constantly emerging viral strains. Our variant analysis of the updated *SARS-CoV-2* variant database reveals a uniform distribution of variants across the genome, with selective enrichment of variants at hotspot regions. Additionally, we extended our analysis to include the emerging strains, B1.1.7, B1.135 and P1, and present a comparative account of recurrent mutations among these strains against the Indian variant pool to determine any pre-existing variants from the novel strains. From the generated database, using IPD 2.0, we further evaluate the clade assessment accuracy and factors affecting the clade prediction accuracy, including genome coverage, number of variants per sample and background mutation rate in the *SARS-CoV-2* genomes.

## Materials and methods

### Implementation of IPD 2.0 and SARS-CoV-2 clade assignment module

IPD 2.0 is implemented using Python 3, and the external tool dependencies are distributed as a pre-installed Conda [5] environment. The detailed installation process, with the pre-compiled reference data for IPD 2.0, can be found at http://ipd.actrec.gov.in/ipdweb/manual.html. The detailed implementation of the variant analysis and pathogen quantification pipeline has been described earlier [3]. In short, the NGS raw data is filtered based on the sequence quality, nucleotide composition and length, criteria. The selected reads align with a primary reference database consisting of human and pathogen (n = 1060) genomes. The pathogen aligned reads are further assessed for specificity using the secondary alignment module. Finally, the aligned reads are subject to variant calling, using a consensus variant calling approach (called by at least two of three variant callers) and normalized quantification (described in detail in [3]). The *SARS-CoV-2* module of IPD 2.0 focusses on the phylogenetic clade analysis and report generation for the *SARS-CoV-2* genomic samples.

For clade assignment to a particular sample, IPD 2.0 uses the sample variants and compares them against the known clade-specific variants described in the NextStrain repository (https://github.com/nextstrain/ncov). The clade-specific variants are henceforth termed informative variants. From the output of the IPD 2.0 variant analysis pipeline, the informative variants are extracted and used to calculate a cumulative clade score of a sample to be of a specific clade. Each informative variant helps assignment of a cumulative clade score for each of the 13 major clades (19A, 19B, 20A, 20B, 20C, 20D, 20E, 20E.EU, 20F, 20G, 20H/501Y.V2, 20I/501Y.V2, 20J/501Y.V2). The following equation defines the cumulative clade score for a sample to be of a particular clade (*C*_*x*_):

1 $$P\left( {C_{x} } \right) = \mathop \sum \limits_{i = 1}^{n} {\raise0.7ex\hbox{$1$} \!\mathord{\left/ {\vphantom {1 {{\mathbb{N}}_{i} }}}\right.\kern-\nulldelimiterspace} \!\lower0.7ex\hbox{${{\mathbb{N}}_{i} }$}}$$

where *1 … n* are the informative variants for a sample, ‘*N*_*i*_’ is the total number of clades (of the 12 known clades) an informative variant may represent, and *P*(*C*_*x*_) is the cumulative clade score that a sample belongs to clade *C*_*x*_. The clade having the maximum score for a sample is assigned (as shown in Eq. 2).

2 $$C_{x} = max\left\{ {P\left( {C_{1} } \right), P\left( {C_{2} } \right) \ldots P\left( {C_{13} } \right)} \right\}$$

The novel variants and the clade assignment are reported in an automated report generated by the *SARS-CoV-2* module described in IPD.

### Automated SARS-CoV-2 variant database generation

IPD 2.0 contains a module to update the variant database of the *SARS-CoV-2* analysis module. The module requires a user to provide a downloaded *SARS-CoV-2* genome sequence in Fasta format (with original headers) from the GISAID database [6]. The metadata about the sequence name, EPI identifier, and isolate collection date is extracted from the header, while entries without EPI IDs are ignored for further processing. Sequences are trimmed at the ends if having 'N' or '–’ characters, and the once having a length less than the user-defined threshold (default = 29,000 bp) or ‘N’ more than the defined threshold (default = 15,000) are filtered out. Snippy [7] based variant calling is performed individually on the filtered genome sequences, using the Wuhan strain (RefSeq ID: NC_045512) as the reference *SARS-CoV-2* genome. Sample-wise variant profiles are created using the annotated TAB files generated by Snippy, and unique mutation profiles are built by selecting the unique representative sample profiles. The representative mutation profile file is tabix [8] indexed and forms the core variant database of IPD 2.0 *SARS-CoV-2* module. The updater module also maintains the version information of the variant database on the user machine by keeping logs of the genome entries added in the database. The pre-compiled reference database contains the variants from genome sequences as of December 28, 2020, which users can be updated by calling the script ‘ipdsarscov2updater.py’, provided with the source package.

### Clade assessment accuracy evaluation and comparison with IPD

To evaluate accuracy of the clade assessment of the *SARS-CoV-2* module of IPD 2.0, dataset was simulated using the sequences downloaded from GISAID, representing clades 19B (EPI_ISL_410535), 20A (EPI_ISL_448260), 20B (EPI_ISL_448264), 20C (EPI_ISL_523229), 20D (EPI_ISL_474965), 20E.EU1 (EPI_ISL_637212), 20F (EPI_ISL_565007), 20G (EPI_ISL_590772) and 20I.50I.V1 (EPI_ISL_728566). Clade assessment of the sequences was performed using the NextClade module of the NextStrain package [9] and used as a truth set. The simulated dataset generated using neat-gen reads [10] consisted of 106 samples. The data was generated with 10×, 20×, and 30× coverage for each clade, having a read length of 101 bp and a varying background mutation burden (0, 0.01%, 0.02% and 0.03%). The run-time comparison between IPD and IPD 2.0 was performed on a 64-bit machine with 14 cores of 2.00 GHz, and the pipeline was run using 5 threads.

## Results and discussion

**Fig. 1:**
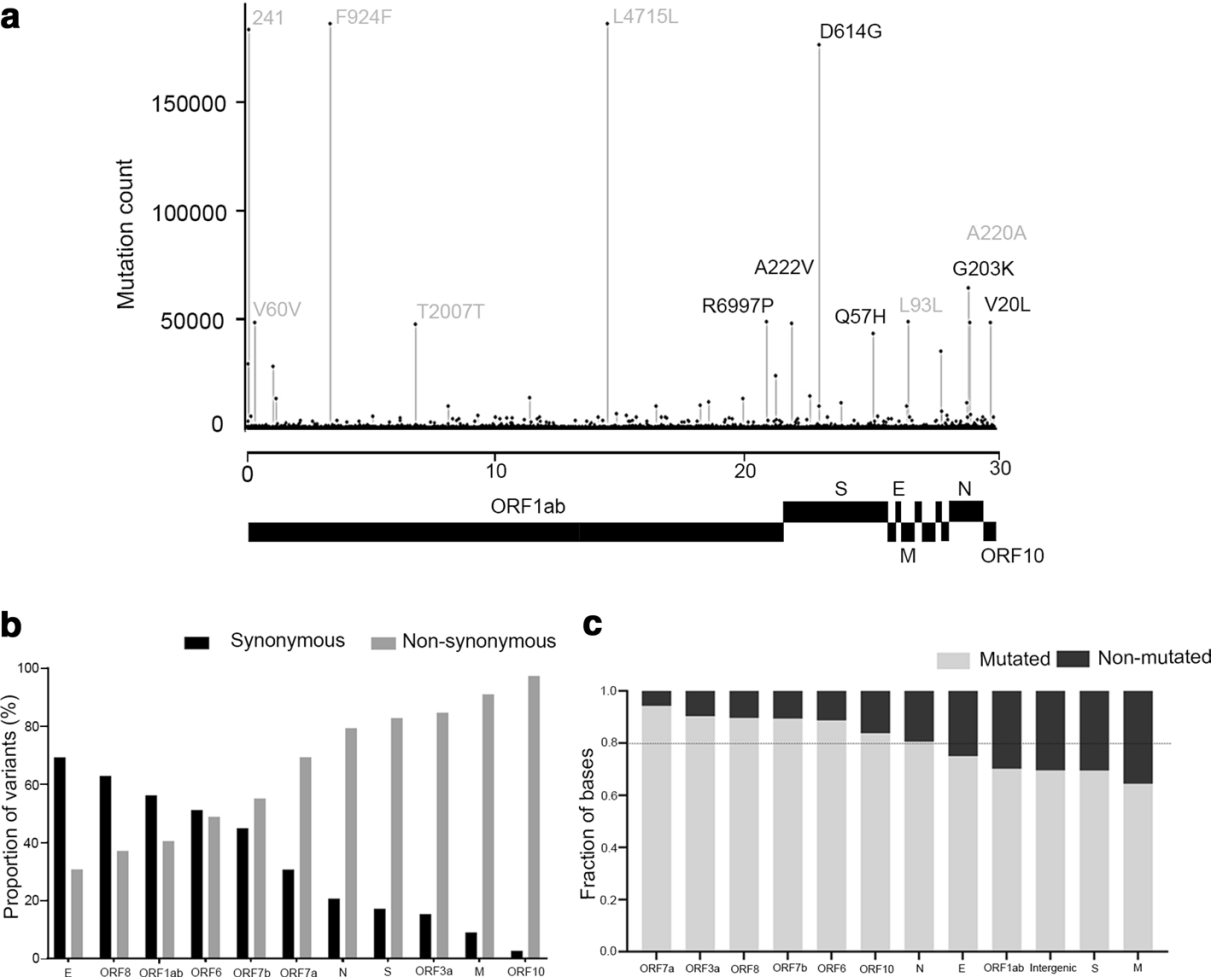
Global distribution and gene-wise mutation analysis of the *SARS-CoV-2* genome mutations. **a** Genomic hotspot mutations (recurrence > 40,000 samples) distribution across the genome. Mutations have been labelled with protein change in the plot. The intergenic and synonymous mutations are colored grey. The gene annotation track on the x-axis is not to scale. **b** Proportion of synonymous and nonsynonymous mutations across all the *SARS-CoV-2* genes, **c** proportion of mutated/non-mutated bases across the *SARS-CoV-2* gene features. The dotted line indicates an average fraction of mutated residues per feature (~ 0.8)

Complete, high coverage *SARS-CoV-2* genome sequences (N = 208,911) from 155 different countries, with length greater than 29,000 bp, were downloaded from the GISAID database [6] (as of December 28, 2020). The genomes were given as input to the *SARS-CoV-2* variant database updater module (*ipdsarscov2updater.py*), which automatically generates the variant database and the representative SARS-CoV-2 mutation profile database used in IPD 2.0. Upon trimming ‘N’ at the ends and filtration of sequences based on length selection (minimum length of 29,000 bp), 200,865 sequences were retained. The mutation analysis of these *SARS-CoV-2* genomes resulted in 2.58 million variants, in which we find 1,004,453 (38.88%) synonymous, 1,327,548 (51.39%) nonsynonymous mutations and 242,631 (9.39%) mutations in the intergenic region comprising of coding 5' and 3'UTRs, indicating a relatively higher representation of nonsynonymous mutations. Among nonsynonymous mutations, missense mutations (49.54%) were more frequent than stop lost (1.17%), stop gain (0.66%) and deletions/ insertions (0.23%). Overall, 6.6 nonsynonymous, 5 synonymous and 1.20 intergenic mutations per sample were observed (Additional file 1: Table S1). From the variant dataset generated, we observed 13 hotspot residues across the *SARS-CoV-2* genome that occur at least in 40,000 samples or more in a non-exclusive manner (Fig. 1a, Additional file 1: Table S2), consistent with the literature [11]. The 13 most recurrent hotspot mutations found comprise 5 synonymous mutations likely affecting mRNA splicing or selection on codon usage bias, stability and folding translation or co-translational protein folding [12–14] remains to be explored.

The variant dataset generated from 200,865 *SARS-CoV-2* genomes was further used to perform a gene-wise mutation analysis. We estimated frequencies for genes with under-sampled synonymous mutations accounting for the individual gene biases. Our analysis revealed that after normalizing for gene length, the S, N, M, ORF7a, and ORF10 viral genes comprised about 21% of the genome, accounts for 54.36% of all *SARS-CoV-2* nonsynonymous mutations (Fig. 1b). Interestingly, S and M genes harbor the least proportion of total variable bases across the *SARS-CoV-2* genome, indicating that the restricted bases undergoing nonsynonymous mutations are under selection, in both the genes (Fig. 1c). The insights of the functional relevance of the different amino acid sites mutated though remain to be established. We also analyzed for variants in the newer *SARS-CoV-2* virus lineage B1.1.7 (clade 20I/501.V1) emerging in the UK [15], B.1.351 (clade 20H/501Y.V2) in South Africa [16], and P.1 (clade 20J/501Y.V3) in Brazil [17] that were found to harbour a total of 32, 25 and 25 median mutations across 13, 82 and 13 samples, respectively, for each lineage (Additional file 1: Tables S3 and S4).

**Fig. 2:**
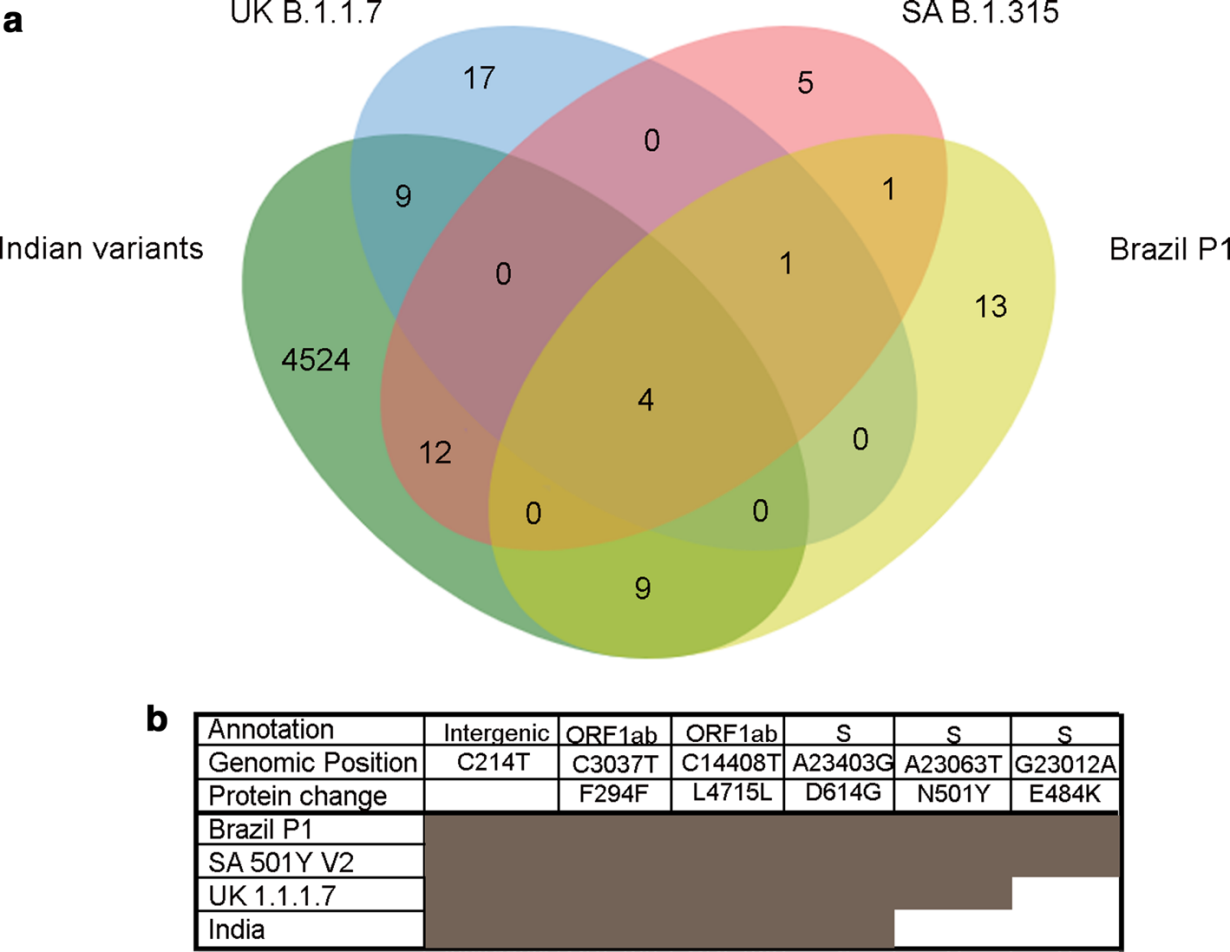
Overlap of variants recurring among the emerging strains (B 1.1.7, B 1.135 and P1) and Indian samples. **a** Variants recurring in at least 50 per cent of analyzed samples are overlapped with variants in Indian samples. **b** Variants common across all the strains, including Indian samples and private clade defining variants in the S protein across the emerging *SARS-CoV-2* strains

A comparative account of variants predominant in the three newer lineages originating from distinct geographical regions along with those reported from India, comprising of 3361 samples with a comparable frequency of nonsynonymous mutations (48.75%) and synonymous mutations (41.45%) (Additional file 1: Table S5), revealed four core common hotspot mutations including D514G mutation in the spike protein and several lineage-restricted unique mutations for each strain (Fig. 2a). Among the three emergent strains, N501Y was found as the root mutation, while the South African and Brazil strain appear to acquire additional lineages specific to E484K mutation within spike protein. Taken together, this suggests a repetitive convergent and adaptive evolution adopted by the distinct lineages (Fig. 2b) that tend to pose a reasonable threat towards the emergence of newer regional variant strains with continued persistence of the pandemic.

**Fig. 3:**
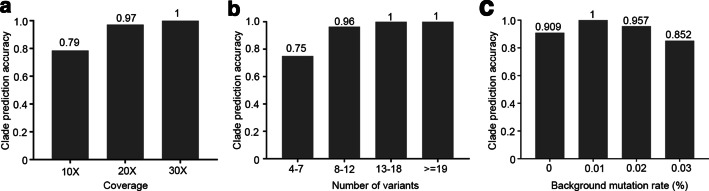
Factors affecting the accuracy of IPD 2.0 *SARS-CoV-2* clade prediction module, **a** clade prediction accuracy based on the samples with different genome coverage, **b** comparison of prediction accuracy based on several variants obtained per sample, **c** variation in the clade prediction accuracy based on the background mutation rate of the *SARS-CoV-2* genomes

With the inclusion of the variant database and revised clade assessment module based on the recent clade nomenclature proposed [4], we benchmarked the *SARS-CoV-2* clade assessment module of IPD 2.0 against the NextStrain clade assignments. We further also evaluated the parameters affecting the clade prediction accuracy of the *SARS-CoV-2* module. For this, we used a simulated dataset for 9 out of 13 major *SARS-CoV-2* clades [18] and generated data using varying genome coverage of 10X, 20X, 30X and background genome mutation rate of 0, 0.01, 0.02, 0.03% per *SARS-CoV-2* genome. The overall clade prediction accuracy of IPD 2.0 *SARS-CoV-2* module, across the 103 (91 true positives, 7 false positives, samples with less than 4 variants were excluded from analysis; n = 5) simulated samples representing 9 different clades of *SARS-CoV-2*, is found to be 92.8% (Additional file 1: Table S5). We further evaluated the effect of factors like coverage, number of variants per sample and background mutation rate on the clade prediction accuracy of IPD 2.0. With increasing coverage of the samples, the prediction accuracy was observed to increase from 80% at 10×, 97% at 20×, to 100% at 30× coverage (Fig. 3a). Similarly, with an increase in the number of variants, the prediction accuracy was found to increase. IPD 2.0 reaches the accuracy of 100% for samples with greater than 12 variants, as seen in Fig. 3b. For the 7 samples (of 98) for which IPD 2.0 assigned incorrect clade, 6 had coverage of 10× and the number of variants per sample ranging within 4–7. This indicates that the lower coverage of the samples resulted in a smaller number of variants from the IPD 2.0 variant analysis pipeline, which affected the clade prediction accuracy. As shown in Fig. 3c, the increasing background mutation rate for the *SARS-CoV-2* genome sequences decreased prediction accuracy. Further, we also compared the run-time usage of IPD 2.0 with its predecessor. The run-time comparison of the desktop version showed that the average time taken to process a sequencing sample is reduced up-to 55.27% in IPD 2.0 (mean time = 63 min) as compared to IPD (mean time = 140.86 min). Similarly, for the SARS-CoV-2 analysis module, there is 81.52% mean run-time reduction (IPD = 11.75, IPD 2.0 = 2.17) (Additional file 2: Figure S1). Parallelization in the variant calling pipeline and indexed data structure of the variant database in the *SARS-CoV-2* module greatly reduced the run-time compared with IPD.

## Conclusion

In summary, we present IPD 2.0, an improved version of our previously reported method [3] for pathogen quantification and variant calling of 1060 infectious pathogens, with a specialized module for *SARS-CoV-2* phylogenetic analysis. In addition to the improvement in the run-time of the variant calling/*SARS-CoV-2* module and clade prediction accuracy (92.8% compared to 77–83% in the case of its predecessor), IPD 2.0 adopts the recently proposed clade nomenclature [4]. The IPD 2.0 package also incorporates an additional module to allow users to update the core genome and variant database of the *SARS-CoV-2* analysis module, making IPD 2.0 uniquely distinct from the other viral genomic data analysis tools and adaptable to the constantly increasing *SARS-CoV-2* genome data in the public repositories. With the evolving landscape of *SARS-CoV-2* mutations and strains world-wide, the dynamic nature of IPD 2.0 makes it a contemporary tool to facilitate genomic surveillance to identify variants involved in breakthrough infections.

## Availability and requirements

- Project name: Infectious Pathogen Detector 2 (IPD 2.0).
- Project home page: http://www.actrec.gov.in/pi-webpages/AmitDutt/IPD/IPD.html and http://ipd.actrec.gov.in/ipdweb/
- Operating system(s): Linux (desktop version).
- Programming language: Python 3.0
- Other requirements: Conda, Tkinter (for GUI).
- License: MIT license.
- Any restrictions to use by non-academics: license needed.

## Supplementary Information

**Additional file 1**. Supplementary Tables (S1–S6).

**Additional file 2**. Supplementary Figure S1. Run time comparison between IPD and IPD 2.0 with simulated amplicon dataset (n = 19), A) variant calling and quantification pipeline run-time comparison B) SARS-CoV-2 module run time comparison

## Data Availability

The Infectious Pathogen Detector 2 (IPD 2.0) has been made freely available to the scientific community as a web-based server at http://ipd.actrec.gov.in/ipdweb/. The desktop version of the IPD 2.0 tool can be downloaded from http://www.actrec.gov.in/pi-webpages/AmitDutt/IPD/IPD.html and pre-build reference files for local desktop version can be found at http://ipd.actrec.gov.in/referencedatabase/data.tar.gz. The user-manual for usage of IPD 2.0 can be found at http://ipd.actrec.gov.in/ipdweb/manual.html. Raw SARS-CoV-2 genome sequences have been obtained from https://www.gisaid.org/, for generation of variant database presented in the study.

## Abbreviations

SARS-CoV-2: Severe acute respiratory syndrome coronavirus 2
NGS: Next generation sequencing
GISAID: Global initiative on sharing all influenza data
IPD: Infectious pathogen detector
