# Supplementary figures and images for: IPD 2.0: To derive insights from an evolving SARS-CoV-2 genome
Source: BMC Bioinformatics. 2021 May 13;22:247. doi: 10.1186/s12859-021-04172-x (PMC8118100; doi:10.1186/s12859-021-04172-x)

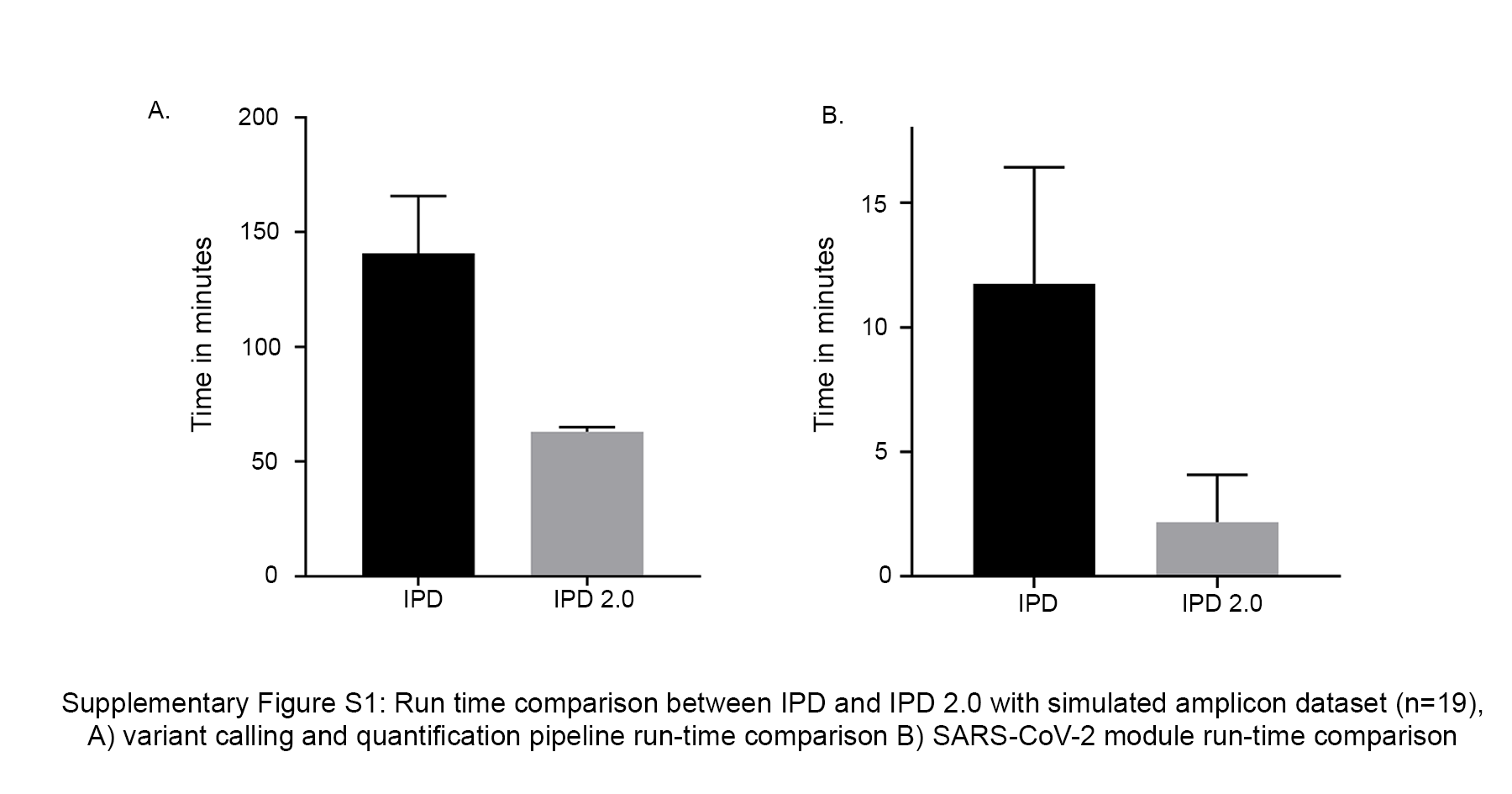

Supplement: Supplementary file 2 — Additional file 2. Supplementary Figure S1. Run time comparison between IPD and IPD 2.0 with simulated amplicon dataset (n = 19), A) variant calling and quantification pipeline run-time comparison B) SARS-CoV-2 module run time comparison [file 12859_2021_4172_MOESM2_ESM.tif]
